# Optimizing complex phenotypes through model-guided multiplex genome engineering

**DOI:** 10.1186/s13059-017-1217-z

**Published:** 2017-05-25

**Authors:** Gleb Kuznetsov, Daniel B. Goodman, Gabriel T. Filsinger, Matthieu Landon, Nadin Rohland, John Aach, Marc J. Lajoie, George M. Church

**Affiliations:** 1 Department of Genetics, Harvard Medical School, Boston, MA USA; 2 Wyss Institute for Biologically Inspired Engineering, Harvard Medical School, Boston, MA USA; 3 Program in Biophysics, Harvard University, Boston, MA USA; 4 Systems Biology Graduate Program, Harvard Medical School, Boston, MA USA; 5 Ecole des Mines de Paris, Mines Paristech, Paris, France

**Keywords:** Genome engineering, Predictive modeling, Synthetic organisms

## Abstract

We present a method for identifying genomic modifications that optimize a complex phenotype through multiplex genome engineering and predictive modeling. We apply our method to identify six single nucleotide mutations that recover 59% of the fitness defect exhibited by the 63-codon *E. coli* strain C321.∆A. By introducing targeted combinations of changes in multiplex we generate rich genotypic and phenotypic diversity and characterize clones using whole-genome sequencing and doubling time measurements. Regularized multivariate linear regression accurately quantifies individual allelic effects and overcomes bias from hitchhiking mutations and context-dependence of genome editing efficiency that would confound other strategies.

**Electronic supplementary material:**

The online version of this article (doi:10.1186/s13059-017-1217-z) contains supplementary material, which is available to authorized users.

## Background

Genome editing and DNA synthesis technologies are enabling the construction of engineered organisms with synthetic metabolic pathways [1], reduced and refactored genomes [2–5], and expanded genetic codes [6, 7]. However, genome-scale engineering can come at the cost of reduced fitness or suboptimal traits [2, 7] caused by design flaws that fail to preserve critical biological features [7, 8], synthesis errors, or collateral mutations acquired during strain construction [6]. It remains challenging to identify alleles that contribute to these complex phenotypes and prohibitive to test them individually. Laboratory evolution has traditionally been used to improve desired phenotypes and navigate genetic landscapes [9]; however, this process relies on mutations that accumulate across the genome and may disrupt synthetic designs or traits not maintained under selection. In contrast, targeted genome engineering can alter the genome at chosen loci and can be used to target many locations simultaneously [10]. Multiplexed editing creates a large pool of combinatorial genomic changes than can be screened or selected to find high-performing genomic designs. However, as the number of targeted loci considered increases, it becomes difficult to interpret the significance of individual changes. There remains a need for a method to rapidly identify subsets of beneficial alleles from a large list of candidates in order to optimize large-scale genome engineering efforts.

**Fig. 1:**
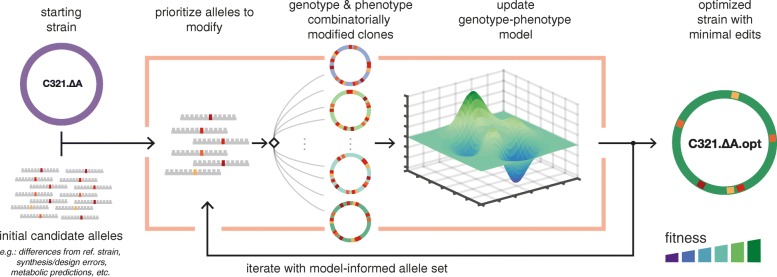
Workflow for improving phenotypes through model-guided multiplex genome editing. First, an initial set of target alleles (hundreds to thousands) is chosen for testing based on starting hypotheses. These targets may be designed based on differences from a reference strain, synthesis or design errors, or biophysical modeling. Multiplex genome editing creates a set of modified clones enriched with combinations of the targeted changes. Clones are screened for genotype and phenotype and predictive modeling is used to quantify allele effects. The workflow is repeated to validate and test new alleles. Beneficial alleles are combined to create an optimized genotype

Leveraging recent improvements in the cost and speed of microbial whole-genome sequencing (WGS), we present a method for identifying precise genomic changes that optimize complex phenotypes, combining multiplex genome engineering, genotyping, and predictive modeling (Fig. 1). Multiple rounds of genome editing are used to generate a population enriched with combinatorial diversity at the targeted loci. Throughout the editing process, clones from the population are subject to WGS and are screened for phenotype. The genotype and phenotype data are used to update a model which predicts the effects of individual alleles. These steps are repeated on a reduced set of candidate alleles informed by the model or on a new set of targets. Finally, the highest impact alleles are rationally introduced into the original organism, minimizing alterations to the organism’s original genotype while optimizing the desired phenotype.

We applied this method to the genomically recoded organism (GRO) C321.∆A, a strain of *E. coli* engineered for non-standard amino acid (nsAA) incorporation [6]. C321.∆A was constructed by replacing all 321 annotated UAG stop codons with synonymous UAA codons and deleting UAG-terminating Release Factor 1. Over the course of the construction process, C321.∆A acquired 355 off-target mutations and developed a 60% greater doubling time relative to its non-recoded parent strain, *E. coli* MG1655. An improved C321.∆A strain would accelerate the pace of research involving GROs and further enable applications leveraging expanded genetic codes, including biocontainment [11, 12], virus resistance [13], and expanded protein properties [14]. We expected that a subset of the off-target mutations caused a considerable fraction of the fitness defect, providing a starting hypothesis for iterative improvement.

## Results

To select an initial set of candidate alleles (Additional file 1: Figure S1), we first used the genome engineering and analysis software *Millstone* [15] to analyze sequencing data from C321.∆A and to identify all mutations relative to the parental strain MG1655. *Millstone* uses SnpEff [16] to annotate affected genes and predicted severity of each mutation. We further annotated each coding mutation with the growth defect of its associated gene’s Keio collection knockout strain after 22 hours in lysogeny broth (LB_22) [17]. Based on this analysis, we identified 127 mutations in proteins and non-coding RNA as the top candidates responsible for fitness impairment. Our candidate alleles included all frameshift and non-synonymous mutations, mutations in non-coding RNA, and synonymous changes in genes with LB_22 < 0.7. We partitioned the targets into three priority categories according to predicted effect (Additional file 2 and Additional file 3).

MAGE introduces combinations of genome edits with approximately 10–20% of cells receiving at least one edit per cycle [10]. To generate a diverse population of mutants enriched for reversions at multiple loci, we performed up to 50 cycles of MAGE in three lineages. The first lineage used a pool of 26 oligonucleotides targeting only the highest category of mutations, the second lineage targeted the top 49 sites, and the third lineage targeted all 127 (Additional file 1: Figure S1).

**Fig. 2:**
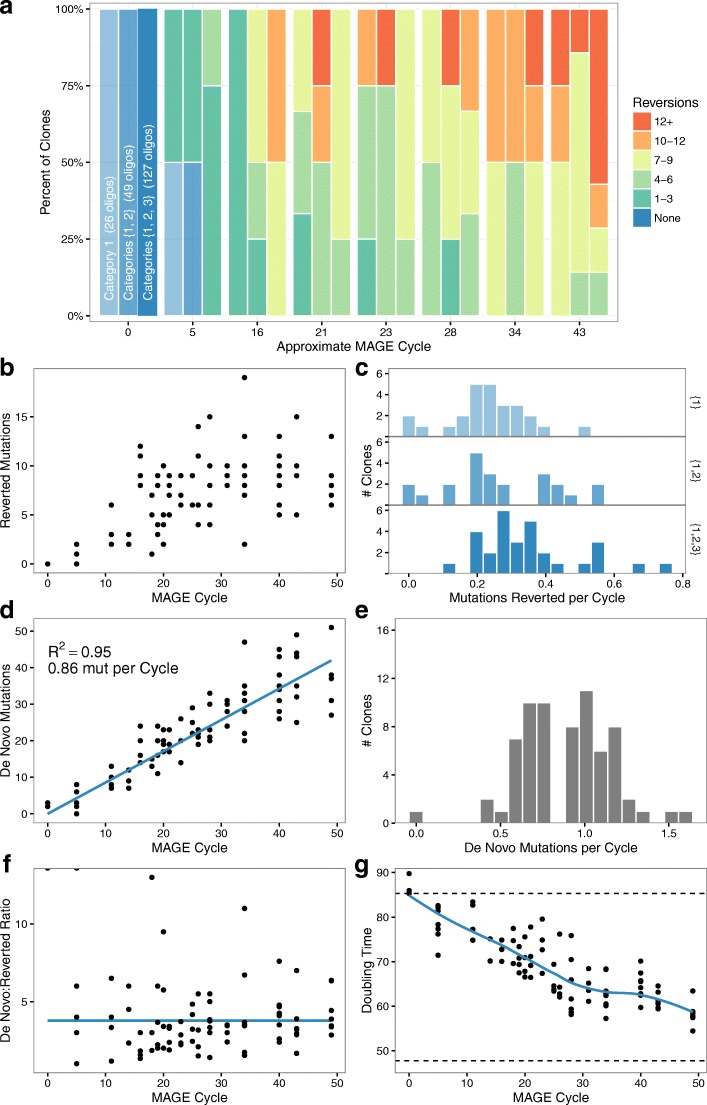
Mutation dynamics over many cycles of MAGE allele reversion. **a** Increase in combinatorial diversity and reversion count vs. number of MAGE cycles. **b** Number of reversions per clone vs. MAGE cycle. **c** The rate of reversions per MAGE cycle among the different allele categories, showing a higher rate per cycle for cells exposed to all 127 oligos. **d** The number of de novo mutations per clone over successive MAGE cycles. **e** Rate of de novo mutations per MAGE cycle. **f** The average ratio between number of de novo mutations and reverted alleles per MAGE cycle remains constant throughout the experiment. **g** Doubling time (min) improvement per clone from the C321.∆A starting strain (*top dotted line*) towards the ECNR2 parent strain (*bottom dotted line*). *Blue line* is a LOESS fit

**Fig. 3:**
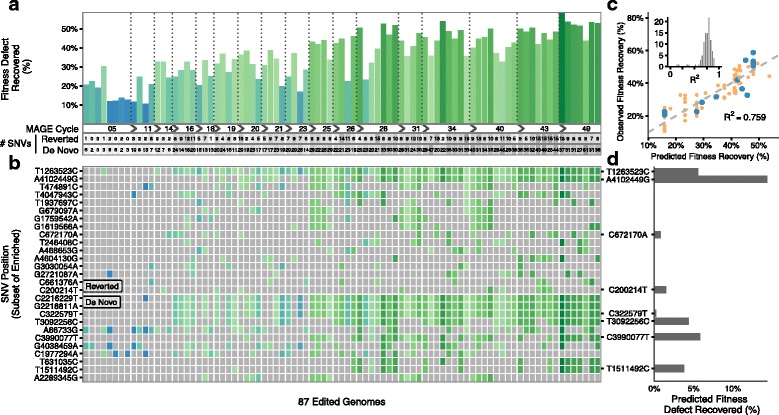
Genotypic and phenotypic diversity in 87 clones sampled across 50 MAGE cycles enabled model-guided prioritization of top single nucleotide variants (SNVs) for further validation. **a** Percent of C321.∆A fitness defect recovered across MAGE cycles (shown with bar color and height). The number of SNVs reverted or introduced are shown below. **b** Presence of targeted reversions and de novo mutations in each clone colored according to fitness. A subset of the most enriched mutations is shown, ordered by enrichment (full dataset available in Additional file 10). **c** Example model fit using top eight alleles as features with 15 samples left out as a test set (*blue* points) and used to evaluate R^2^. Training points are plotted in *orange*. The inset shows distribution of R^2^ values for 100 different simulations with 15 random samples left out to calculate R^2^ for each. Example fit was chosen to exemplify a median R^2^ value from this distribution. **d** Average model fit coefficients for top eight alleles assigned non-zero values over repeated cross-validated linear regression (see “Methods”) indicate their predicted contribution to fitness improvement

We sampled a total of 90 clones from multiple time points and lineages during MAGE cycling, including three separate clones of the starting strain. We then performed WGS and measured doubling time for each clone. *Millstone* was used to process sequencing data and to report variants for all 90 samples in parallel [15]. We observed fitness improvement across all three lineages with a diversity of genotypes and fitness phenotypes across the multiple time points (Figs. 2 and 3a, b). Clones selected from the final time point recovered 40–58% (mean 49%) of the fitness defect compared to MG1655 and had 5–15 (mean 10.2) successfully reverted mutations. Of the 127 targeted mutations, 99 were observed in at least one clone, with as many as 19 successful reversions in a clone from the 127-oligo lineage. Additionally, we observed 1329 unique de novo mutations across all clones (although only 135 were called in more than one clone), accumulating at a rate of roughly one per MAGE cycle in each clone (Fig. 2d, e). This elevated mutation rate was caused by defective mismatch repair (Δ*mutS*), which both increases MAGE allele replacement frequency and provides a source of new mutations that could improve fitness.

The combinatorial diversity produced by sampling at regular intervals between consecutive rounds of multiplex genome engineering generates a dataset well suited for analysis by linear regression (Additional file 1: Supplementary Note 3). Initially, we made a simplifying assumption that doubling time is determined by the independent effects of individual alleles and employed a first-order multiplicative model that predicts doubling time based on allele occurrence (see “Methods” and Additional file 1: Supplementary Note 1). As model features, we considered the 99 reversions and 135 de novo mutations that occurred in at least two clones. Multivariate linear regression was used to fit the model, with feature coefficients indicating the predicted effect of the respective allele. We considered several priors in selecting our specific modeling strategy: (1) we expected a small number of alleles to contribute significantly to fitness improvement; (2) the continuous passaging nature of our experiment may allow hitchhiker alleles to become associated with causal alleles. Thus, we chose to use elastic net regularization [18], which adds a weighted combination of L1 and L2 terms to the objective function. To limit overfitting, we performed multiple rounds of k-fold cross-validation (k = 5) and selected alleles that were assigned a non-zero coefficient on average. The analysis of the data obtained over 50 cycles of MAGE identified four targeted reversions and four de novo mutations that had the greatest putative effect on fitness (Fig. 3c, d and Additional file 4).

To validate the eight alleles prioritized in the 50-cycle MAGE experiment, we performed nine cycles of MAGE using a pool of eight oligos (Additional file 4) applied to the starting C321.∆A strain. We then screened each clone using multiplex allele-specific colony polymerase chain reaction (MASC-PCR) (see “Methods”) and measured doubling time (Additional file 1: Figure S2). Modeling revealed strong effects for two reversions (*hemA*-T1263523C and *cpxA*-A4102449G) and one de novo mutation (*cyaA*-C3990077T), along with weaker effects for two additional reversions (*bamA*-C200214T and *leuS*-C672170A). These mutations are discussed in Additional file 1: Supplementary Note 2. A clone with all five of these mutations was isolated and measured to have recovered 51% of the fitness defect exhibited by C321.∆A. The three remaining de novo mutations did not show evidence of improving fitness despite being highlighted in the initial modeling, illustrating the importance of subsequent validation of model-selected alleles.

To identify mutations that further improved the fitness of C321.∆A, we extended our search to off-target mutations occurring in regulatory regions using smaller pool sizes. We identified seven non-coding mutations predicted to disrupt gene regulation [8] (see “Methods” and Additional file 5). Applying nine rounds of MAGE followed by linear modeling identified the reversion C49765T, a mutation in the -35 box of the *folA* promoter, which recovers a predicted 27% of the fitness defect (Additional file 1: Figure S3).

To test whether any of the designed UAG-to-UAA mutations caused a fitness defect in the C321 background, we followed the same procedure with 20 previously recoded UAA codons predicted to have a potentially disruptive effect (Additional file 6). We tested reversion back to UAG in a *prfA* ^+^ variant of C321 capable of terminating translation at UAG codons. We observed no evidence of a beneficial fitness effect from any individual UAA-to-UAG reversion.

**Fig. 4:**
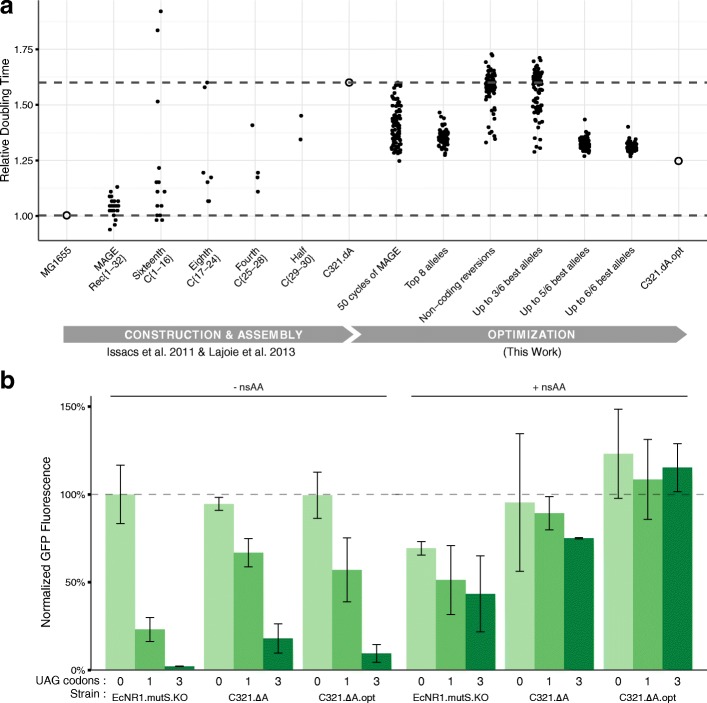
Construction and characterization of final strain C321.∆A.opt. **a** Doubling time of clones isolated during construction and optimization of C321.∆A. Strain C321.∆A.opt was constructed in seven cycles of MAGE in batches of up to three cycles separated by MASC-PCR screening to pick clones with the maximum number of alleles converted (see “Methods”). The two *dotted horizontal lines* correspond to the relative doubling times for the original GRO and the wild-type strain. **b** Testing nsAA-dependent protein expression using the nsAA p-acetyl-L-phenylalanine (pAcF) in sfGFP variants with 0, 1, or 3 residues replaced with UAG codons. Normalized GFP fluorescence was calculated by taking the ratio of absolute fluorescence to OD600 of cells suspended in phosphate buffered saline (PBS) for each sample and normalizing to the fluorescence ratio of non-recoded strain EcNR1.mutS.KO expressing 0 UAG sfGFP plasmid

Finally, we used MAGE to introduce the best six mutations (Additional file 7) into the original C321.∆A strain (see “Methods”), creating an optimized strain C321.∆A.opt that restores 59 +/– 11% of the fitness defect in C321.∆A (Fig. 4a). This rationally designed strain recovered the same amount of fitness as the fastest clones obtained through 50 rounds of MAGE and substantial passaging, which resulted in 6–13 reversions and 31–38 de novo mutations (Fig. 4a). WGS of the final strain confirmed that no UAG codons were reintroduced. Nine additional de novo mutations arose, but these are predicted to have a neutral effect (Additional file 8). We characterized UAG-dependent incorporation of the nsAAs p-acetyl-L-phenylalanine (pAcF) in C321.∆A.opt using sfGFP variants with 0, 1, and 3 residues replaced by the UAG codon and confirmed that C321.∆A.opt maintains nsAA-dependent protein expression (Fig. 4b). C321.∆A.opt has been deposited at AddGene (Bacterial strain #87359).

**Fig. 5:**
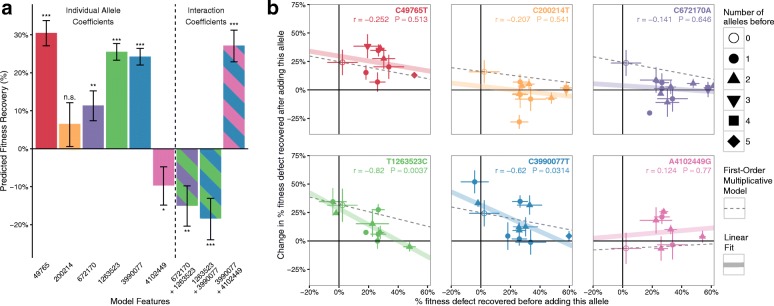
Interactions among top six alleles show evidence of epistasis. Genotypes and fitness measurements were obtained from 359 intermediate clones generated during the construction of the final strain containing the six best alleles (Additional file 7). Each clone was genotyped using MASC-PCR and doubling time was measured during allele validation experiments and final strain construction. **a** Individual model coefficients for the top six alleles, as well as three significant interaction terms identified during combinatorial construction. These values are from a linear model with interaction terms between each pair of alleles. The *error* *bars* signify the standard error of the mean of the model coefficients and the significance codes for a non-zero effect size are: *** *p* < 0.001, ** 0.001 ≤ *p* < 0.01, * 0.01 ≤ *p* < 0.05, *n.s.* not significant. All three interactions coefficients remain significant after a family-wise error rate (FWER) of $\upalpha$ = 0.05/C(6,2) = 0.003. **b** Each data point represents the amount of fitness recovered when adding the allele specified to an identical starting genotype background. *Horizontal error bars* correspond to the standard deviation of fitness defect among all clones with this starting genotype. *Vertical error bars* represent the standard deviation of all differences between clones with and without the respective allele. For each plot, the thick *colored line* represents a simple linear fit through the points, corresponding to the *r* and *p* values given in each plot. The *dotted line* corresponds to the predicted fit for a simple multiplicative model of fitness where the allele always recovers a constant percent of the remaining fitness defect regardless of the background. For all alleles except A4102449G (*pink*), adding the allele to C321 showed a recovery of the fitness defect (>0 on the y axis), with the percentage of defect recovered decreasing as other alleles are also reverted, consistent with a first-order multiplicative model. In some cases, the fitness improvement drops more rapidly than predicted by the multiplicative model (i.e. points below the *dotted lines*), suggesting diminishing returns epistasis. This is supported by the negative-coefficient interaction terms in panel (**a**). In the case of A4102449G there appears to be a negative effect with the mutation alone, but an increase in the presence of other alleles, suggesting possible sign epistasis

To address the remaining fitness defect, we first examined potential interactions among the six alleles identified. We characterized the fitness of 359 clones with intermediate genotypes generated during the construction of the final strain (Fig. 4a). We applied linear regression with higher order interaction terms (Fig. 5a) and observed that combinations of mutations tended to produce diminishing returns [19], suggesting that additional beneficial alleles would only contribute marginally to fitness (Fig. 5b). To evaluate the possibility that our modeling procedure did not detect all effects among alleles tested, we performed in silico simulations of a simplified version of our experiment (Additional file 1: Supplementary Note 3) and investigated our ability to detect fitness effect with varying numbers of underlying causal mutations. We found that in the idealized case of no epistasis, we would detect over 90% of total fitness effect given our experimental design parameters (Additional file 1: Figure S4e). A set of relatively weaker mutations may contribute to the remaining fitness defect, although we cannot exclude the possibility that the combination of 321 designed UAG-to-UAA mutations contributes to the global defect as well.

## Discussion and conclusion

In summary, we used an iterative strategy of multiplex genome engineering and model-guided feature selection to converge on six alleles that together recover 59% of the fitness defect in C321.∆A relative to its wild-type ancestor. This method allowed us to quantify the effects of hundreds of individual alleles and then rationally introduce only the minimal set of beneficial genetic changes, reducing unintended effects from additional off-target mutations.

Our approach reveals several problems inherent to simply using enrichment to rank allelic effect. Our data show that alleles enriched over rounds of selection are not necessarily well-correlated with fitness. Allele enrichment may be affected by differences in editing efficiency, competition among beneficial alleles through clonal interference, and genetic drift. Combinatorial targeted editing overcomes these obstacles by allowing the measurement of each allele in many genetic backgrounds, so that linear modeling can quantify its average individual effect.

Further, measuring mutation effects in multiplex makes it experimentally tractable to explore a much larger set of mutations. We observed evidence of positive epistatic interactions between some alleles (Fig. 5a, left), which would be harder to identify through singleplex editing strategies. These findings demonstrate the utility of multiplex genome engineering and predictive modeling for studying epistasis.

A similar model-guided approach could be used to augment other multiplex genome modification techniques, including yeast oligo-mediated genome engineering [19] or multiplex CRISPR/Cas9-based genome engineering in organisms that support homology-directed double-stranded break repair [20, 21]. Biosensors tied to selections or screens [22] can extend this method to optimize biosynthetic pathways in addition to fitness. The rapidly declining cost of multiplex genome sequencing [23] will allow this method to scale to thousands of whole genomes, increasing statistical power and enabling the use of more complex models. While we use column-synthesized oligos in this study, chip-based oligo synthesis enables scaling up the number of genomic sites targeted, allowing thousands of alleles to be tested simultaneously [24–26]. Our simulations suggest that the predictive power of this method can support larger number of mutations than we tested with a modest increase in genomes sampled (Additional file 1: Figure S4d). Finally, making genomic changes trackable [27–29] for targeted sequencing could further increase the economy, speed, and throughput of this approach.

Efficiently quantifying the effects of many alleles on complex phenotypes is critical not only for tuning synthetic organisms and improving industrially relevant phenotypes, but also for understanding genome architecture. While our method is used here to identify and repair detrimental alleles to improve fitness, it will also enable rapid prototyping of alternative genome designs and interrogation of genomic design constraints. Iteratively measuring and modeling the effects of large numbers of combinatorial genomic changes in parallel is a powerful approach to navigate and understand genotype-phenotype landscapes.

## Methods

### Media and reagents

All experiments were performed in LB-Lennox (LBL) medium (10 g/L bacto tryptone, 5 g/L sodium chloride, 5 g/L yeast extract) with pH adjusted to 7.45 using 10 M NaOH. LBL agar plates were made from LBL plus 15 g/L Bacto Agar. Selective agents were used at the following concentrations: carbenicillin (50 μg/mL), chloramphenicol (20 μg/mL), gentamycin (5 μg/mL), kanamycin (30 μg/mL), spectinomycin (95 μg/mL), and SDS (0.005% w/v).

### Strains

The construction and genotype of engineered *E. coli* strain C321.∆A was previously described in detail [6]. Here, before improving fitness, we constructed strain *C321.*∆*A.mutSfix.KO.tolCfix.*∆*bla:E* by further modifying C321.∆A to introduce the following changes: (1) the *mutS* gene was reinserted into the C321.∆A strain in its original locus and MAGE was used to disable the gene by introduction of two internal stop codons and a frameshift; and (2) the carbenicillin-resistance marker *bla* was swapped for gentamicin resistance marker *aacC1* in the lambda red insertion locus. Several control assays were performed in EcNR1.mutS.KO, a non-recoded by MAGE-enabled strain similar to EcNR2 [10]. All genomic positions reported in the manuscript are in the frame of MG1655 K12 (Genbank accession NC_000913.2). The final C321.∆A.opt strain has been deposited at AddGene (Bacterial strain #87359).

### *Millstone*, software for multiplex genome analysis and engineering

*Millstone* [15] was used throughout the project to rapidly process WGS data and identify variants in each sample relative to the reference genome, to explore variant data, and to design oligonucleotides for MAGE. The *Millstone* analysis pipeline takes as input raw FASTQ reads for up to hundreds of clones and a reference genome as Genbank or FASTA format. The software then automates alignment of reads to the reference using the Burrows-Wheeler Aligner (BWA-MEM) followed by single nucleotide variant (SNV) calling using Freebayes. *Millstone* performs variant calling in diploid mode, even for bacterial genomes. This helps account for paralogy in the genome and results in mutation calls being reported as “homozygous alternate” (strong wild-type), “heterozygous” (marginal), or wild-type, along with an “alternate fraction” (AF) field that quantifies the fraction of aligned reads at the locus showing the alternate allele. Marginal calls were inspected on a case-by-case basis using *Millstone*’s JBrowse integration to visualize raw read alignments. *Millstone* provides an interface for exploring and comparing variants across samples. After initial exploration and triage in *Millstone*, we exported the variant report from *Millstone* for further analysis and predictive modeling. In follow-up analysis, we determined empirically that 0.1 < AF < 0.7 indicated a variant call was marginal in our data.

### Identifying off-target mutations for reversions

For the 50-cycle MAGE experiment, we considered only mutations occurring in regions annotated as coding for a protein or functional RNA. Using *Millstone* annotations of predicted effect and Keio knock-out collection annotation of essentiality [17], we defined three priority categories according to expected effect on fitness (Additional file 2). A total of 127 targets were allocated to the three categories to be used for the 50-cycle MAGE experiment.

For a separate experiment, off-target mutations in regulatory regions were selected based on the criteria of predicted regulatory disruption of essential genes and several non-essential genes with particularly strong predicted disruption. Regulatory disruption was determined based on calculating change in 5′ messenger RNA (mRNA) folding or ribosome binding site (RBS) motif strength for mutations occurring up to 30 bases upstream of a gene. We calculated mRNA folding and RBS motif disruption as described in [8]. Briefly, the minimum free energy (MFE) of the 5-prime mRNA structure was calculated using Unafold’s hybrid-ss-min function [30] (T = 37 °C), taking the average MFE between windows of RNA (–30, +100) and (–15, +100) relative to the start codon of the gene. Mutations that caused a change in MFE of the mRNA of over 10% relative to the wild-type context were prioritized for testing. To predict RBS disruption, the Salis RBS Calculator [31] was provided with sequence starting 20 bases upstream of the gene ATG and including the ATG. Mutations that caused a greater than tenfold change in predicted expression were included for testing. Finally, we also considered mutations that overlapped promoters of essential genes based on annotations from RegulonDB [32].

The 20 UAG-reversion targets were chosen when UAGs occurred in essential genes, introduced non-synonymous changes in overlapping genes, or disrupted a predicted regulatory feature as above.

### Multiplex automated genome engineering

Single-stranded DNA oligonucleotides for MAGE were designed using *Millstone*’s optMAGE integration (https://github.com/churchlab/optmage). Oligos were designed to be 90 bp long with the mutation located at least 20 bp away from either end. We used the C321.∆A reference genome (Genbank accession CP006698.1) for oligo design to avoid inadvertently reverting intentional UAG-to-UAA changes. OptMAGE avoids strong secondary structure (< −12 kcal mol − 1) and chooses the sense of the oligo to target the lagging strand of the replication fork [10]. Phosphorothioate bonds were introduced between the first and second and second and third nucleotides at the 5-prime end of each oligo to inhibit exonuclease degradation [10]. All DNA oligonucleotides were purchased with standard purification and desalting from Integrated DNA Technologies and dissolved in dH20.

MAGE was performed as described in [10], with the following specifications: (1) cells were grown at 34 °C between cycles; (2) we noted that C321.∆A exhibits electroporation resistance so a voltage of 2.2 kV (BioRad GenePulser, 2.2 kV, 200 ohms, 25 μF was used for cuvettes with 1 mm gap) was chosen based on optimization using a lacZ blue-white screen; and (3) total concentration of the DNA oligonucleotide mixture was 5 μM for all electroporations (i.e. the concentration of each oligo was adjusted depending on how many oligos were included in the pool).

The 50-cycle MAGE experiment was carried out in three lineages, with oligo pool sizes of 26, 49, and 127 consisting of oligos from priority categories {1}, {1,2}, and {1,2,3}, respectively (Additional file 2). Note that we originally began with just two pools—the top 26 and all 127 oligos—but after five MAGE cycles the lineage exposed to all 127 oligos was branched to have a separate lineage with only the 49 category {1, 2} oligos in order to obtain more enrichment of the higher priority targets. In order to prevent any population from acquiring permanent resistance to recombination, we toggled the dual-selectable marker tolC at recombinations 23, 31, and 26 for the three lineages, respectively, as described in [32]. Briefly, an oligo introducing an internal stop codon in *tolC* was included in the recombination, and after at least 5 h of recovery, cells were selected in media containing colicin E1, which is toxic in *tolC* ^*+*^ *E. coli*. In the subsequent recombination, an oligo restoring *tolC* function was included in the pool after which cells were selected in the presence of 0.005% SDS (w/v).

Validation MAGE experiments composed of ten or fewer oligos were carried out for up to nine MAGE cycles, as we expected adequate diversity based on previous experience with MAGE efficiency.

### Whole-genome sequencing

Genomic DNA (gDNA) preparation for WGS of 96 clones (only 87 considered in manuscript because sequencing analysis revealed that nine cultures were polyclonal) was performed as in [33]. Briefly, gDNA was prepared by shearing using a Covaris E210 AFA Ultrasonication machine. Illumina libraries were prepared for pooled sequencing as previously described [34]. Barcoded Illumina adapters were used to barcode each strain in a 96-well plate. All 96 genomes were sequenced together on a single lane of a HiSeq 2500 PE150 (Additional file 9). Alternative inexpensive WGS library preparation methods have since become available [23].

WGS data were processed to identify clonal genotypes in *Millstone* and then exported for further analysis (Additional file 10). Demultiplexed.fastq reads were aligned to the MG1655 reference genome. SNVs were reported with *Millstone*, as described above. During analysis, marginal calls were visually confirmed by examining alignments using *Millstone*’s JBrowse integration.

### MASC-PCR

MASC-PCR was used to assess successful reversions in validation experiments of ten or fewer targeted mutations and typically performed for 96 clones in parallel. The protocol was performed as previously described [6]. Briefly, two separate PCRs, each interrogating up to ten positions simultaneously, were performed on each clone to detect whether the C321.∆A or reverted allele was present at each position. For each position, the two reactions shared a common reverse primer but used distinct forward primers differing in at least one nucleotide at the 3′ end to match the SNV being assayed specifically. Positive and negative controls were included when available to aid in discriminating cases of non-specific amplification.

### Measuring fitness

Fitness was determined from kinetic growth (OD600) on a Biotek H-series plate reader. Cells were grown at 34 °C in 150 μL LBL in a flat-bottom 96-well plate at 300 rpm linear shaking. To achieve consistent cell state before reading, clones were picked from agar plates or glycerol, grown overnight to confluence, passaged 1:100 into fresh media, grown again to mid-log (~3 h), and passaged 1:100 again before starting the read. OD measurements were recorded at 5-min intervals until confluence. Doubling times were calculated according to t_double_ = c * ln(2)/m, where c = 5 min per time point and m is the maximum slope of ln(OD600). The maximum slope was determined using a sliding window linear regression through eight contiguous time points (40 min) points rather than between two predetermined OD600 values because not all of the growth curves were the same shape or reached the same max OD600. The script used for analyzing doubling time is available at https://github.com/churchlab/analyze_plate_reader_growth.

### Predictive modeling of allele causality

Choosing alleles for subsequent validation was framed as a feature selection problem. We used predictive modeling to prioritize features. Both targeted reversions introduced by MAGE and de novo mutations were considered.

For most analyses, we used a first-order multiplicative allele effect model, where each allele (reversion or de novo mutation) is represented by a single feature and the fitted coefficient corresponding to that feature represents the allele’s effect on doubling time. To find coefficient values, we fit a linear model where genotypes (WGS or MASC-PCR) predict the logarithm of doubling time. Alleles corresponding to features with the most negative coefficients were selected for validation in smaller sets. An additive model was also tested and yielded similar results, as previously noted by others [19].

While we anticipated the possibility of epistatic effects among alleles tested, a first-order model of the 50-cycle MAGE experiment already had 239 features (99 reversions + 140 de novos observed at least twice) and 87 samples, so we omitted higher-order interaction terms to avoid overfitting due to model complexity. We discuss implications of this independence assumption and other details of our allele effect modeling strategy in Additional file 1: Supplementary Note 1.

Elastic net regularization [18], which includes both L1 and L2 regularization penalties, was used in model-fitting. L1 regularization enforces sparsity, capturing the assumption that a handful of alleles will explain a majority of the fitness effect. L2 regularization prevents any one of a subset of highly correlated alleles from dominating the effect of those alleles, balancing the tendency of L1 to drop subsets of highly co-occurring alleles.

Accordingly, the elastic net loss function used follows from Zou and Hastie [18]:

$$ L\left({\lambda}_1,{\lambda}_2,\beta \right)={\left| y- X\beta \right|}^2+{\lambda}_1{\left|\beta \right|}_1+{\lambda}_2{\left|\beta \right|}^2 $$

where

$$ \begin{array}{l}{\left|\beta \right|}_1={\displaystyle \sum_{j=1}^p}\left|{\beta}_j\right|\\ {}{\left|\beta \right|}^2={\displaystyle \sum_{j=1}^p}{\beta}_j^2\end{array} $$

And the coefficients were estimated according to:

$$ \widehat{\beta}= argmi{n}_{\beta}\left( L\left({\lambda}_1,{\lambda}_2,\beta \right)\right) $$

Elastic net regression was performed using the ElasticNetCV module from scikit-learn [35]. This module introduces the hyperparameters alpha = *λ*_1_ + *λ*_2_ and $11\_\mathrm{ratio}=\frac{\lambda_1}{\lambda_1,+{\lambda}_2}$ and uses k-fold cross validation (k = 5) to identify the best choice of hyperparameters for a given training dataset. We specified the range of l1_ratio to search over as [0.1, 0.3, 0.5, 0.7, 0.9, 0.95, 0.99, 1], which tests with higher resolution near L1-only penalty. This fits our hypothesis that a small number of mutations are responsible for a majority of the fitness effect. For alpha, we followed the default of allowing scikit-learn to search over 100 alpha values automatically computed based on l1_ratio.

To avoid overfitting due to the undersampled nature of the data in the 50-cycle MAGE experiment, we performed 100 repetitions of scikit-learn’s cross-validated elastic net regression procedure, and for each repetition, we randomly held out 15 samples that could be used to evaluate the model fit by that iteration. The model coefficient for each allele was then calculated as the weighted-average across all 100 repetitions using the prediction score on the 15 held-out samples as the weighting factor. Only model coefficients with a negative value (some putative fitness improvement) were considered in a second round of 100 repeats of cross-validated elastic net regression, again with 15 samples held out in each repeat to evaluate the model fit. The weighted-average coefficient values over this second set of 100 repetitions were used to determine the top alleles for experimental validation in a nine-cycle MAGE experiment. While this method reproducibly reported the alleles *hemA*-T1263523C, *cpxA*-A4102449G, and *cyaA*-C3990077T, alleles with weaker predicted effects were detected more stochastically, depending on the randomized train-test split, even with 100 repetitions. We expect that sequencing additional clones, as well as further tuning of our modeling method for detecting weak effects may be warranted in future studies.

To evaluate the results of the nine-cycle MAGE validation experiments, we used unregularized multivariate linear regression. With ten or fewer parameters and ~90 clones, only a single iteration of cross-validated regression applied to the full dataset was required to assign predicted effects without requiring the testing of individual alleles.

Elastic net-regularized multivariate regression was compared to univariate linear regression for our data (Additional file 1: Supplementary Note 1, Additional file 11).

### Final strain construction

C321.∆A.opt was constructed by adding the six alleles identified by the optimization workflow (Additional file 7) to C321.∆A.mutSfix.KO.tolCfix.∆bla:E. A total of seven cycles of MAGE were required, with a MASC-PCR screening step every three cycles to select a clone with the best genotype so far (Fig. 3a), minimizing the total number of cycles required. Three cycles of MAGE were performed using oligos targeting all six alleles. Ninety-six clones were screened by MASC-PCR, and one clone with 3/6 alleles (C49765T, T1263523C, A4102449G) was chosen for the next round of MAGE. Three more rounds of MAGE were performed on top of the clone with 3/6 alleles using only the three remaining oligos. MASC-PCR identified a clone with 5/6 alleles (C49765T, C200214T, C672170A, T1263523C, A4102449G). One more round of MAGE was performed using the remaining oligo and a clone with all six alleles was obtained. Additional off-target mutations acquired during construction as identified by whole genome sequencing of the final clone are listed in Additional file 8.

### Characterizing non-standard amino acid incorporation

nsAA incorporation was measured as previously described [6]. 1-UAG-sfGFP, and 3-UAG-sfGFP reporters were produced by PCR mutagenesis from sfGFP (Additional file 1: Supplementary Note 4), and isothermal assembly was used to clone 0-UAG-sfGFP (unmodified sfGFP), 1-UAG-sfGFP, and 3-UAG-sfGFP into the pZE21 vector backbone [36]. We used the pEVOL-pAcF plasmid to incorporate the non-standard amino acid p-acetyl-L-phenylalanine. Reagents were used at the following concentrations: anhydrotetracycline (30 ng/μL), L-arabinose (0.2% w/v), pAcF (1 mM).

## Additional files

Additional file 1: Supplementary Figures, Notes, and Table Legends [37–45]. (PDF 1142 kb)

Additional file 2: Table S1. Prioritized coding reversion categories. (XLSX 36 kb)

Additional file 3: Table S2. Table of 127 mutations targeted in the 50-cycle MAGE experiment. (XLSX 50 kb)

Additional file 4: Table S3. Top mutations from MAGE cycling. (XLSX 37 kb)

Additional file 5: Table S4. List of non-coding mutations tested. (XLSX 8 kb)

Additional file 6: Table S5. List of amber reversions tested. (XLSX 9 kb)

Additional file 7: Table S6. List of top six mutations used to create C321.DA.opt. (XLSX 36 kb)

Additional file 8: Table S7. List of additional mutations in C321.DA.opt. (XLSX 39 kb)

Additional file 9: Table S8. Cost analysis. (XLSX 35 kb)

Additional file 10: Table S9. Mutations from whole genome sequencing data for 90 clones from 50-cycle MAGE experiment. (XLSX 12780 kb)

Additional file 11: Table S10. Comparison to univariate model (GWAS). (XLSX 45 kb)
