# Supplementary material for: Optimizing complex phenotypes through model-guided multiplex genome engineering
Source: Genome Biol. 2017 May 25;18:100. doi: 10.1186/s13059-017-1217-z (PMC5445303; doi:10.1186/s13059-017-1217-z)
Supplement: Supplementary file 1 — Supplementary Figures, Notes, and Table Legends [37–45]. (PDF 1142 kb) [file 13059_2017_1217_MOESM1_ESM.pdf]

Supplementary Figures, Notes, and Table Legends

Optimizing complex phenotypes through model-guided multiplex genome engineering

Gleb Kuznetsov[1,2,3,6], Daniel B. Goodman[1,2,6], Gabriel T. Filsinger[1,2,4,6], Matthieu Landon[1,4,5], Nadin Rohland[1], John Aach[1], Marc J. Lajoie[1,2,7], George M. Church[1,2,7]

[1]Department of Genetics, Harvard Medical School, Boston, Massachusetts, USA. [2]Wyss Institute for Biologically Inspired Engineering, Harvard Medical School, Boston, Massachusetts, USA. [3]Program in Biophysics, Harvard University, Boston, Massachusetts, USA. [4]Systems Biology Graduate Program, Harvard Medical School, Boston, Massachusetts, USA. [5]Ecole des Mines de Paris, Mines Paristech, Paris, France. [6]These authors contributed equally to this work. [7]Correspondence should be addressed to M.J.L. (mlajoie@uw.edu) or G.M.C. (gchurch@genetics.med.harvard.edu).

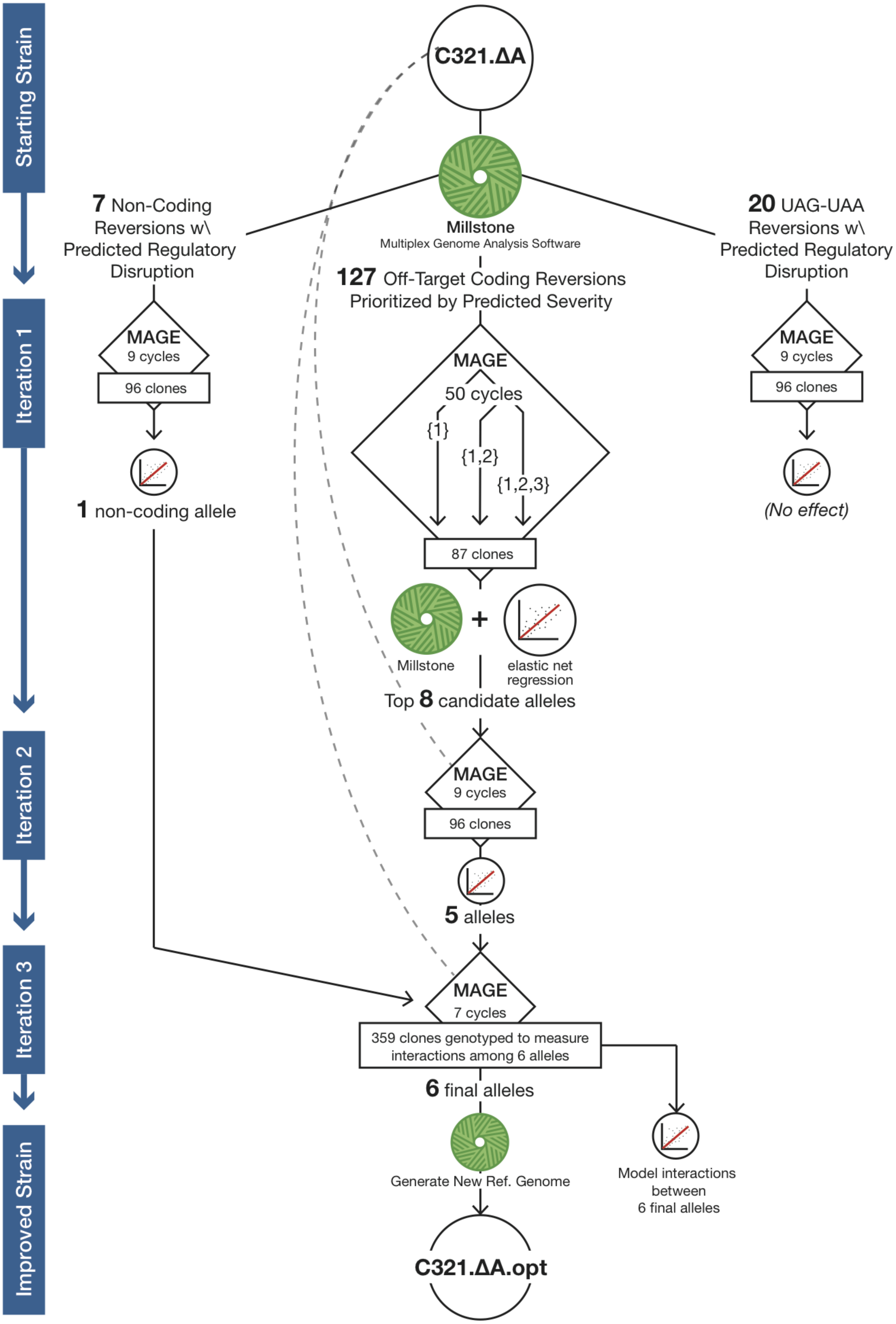
Starting Strain
Iteration 1
Iteration 2
Iteration 3
Improved Strain
C321.ΔA
Millstone
Multiplex Genome Analysis Software
7 Non-Coding Reversions w\ Predicted Regulatory Disruption
20 UAG-UAA Reversions w\ Predicted Regulatory Disruption
127 Off-Target Coding Reversions Prioritized by Predicted Severity
MAGE
9 cycles
96 clones
1 non-coding allele
MAGE
50 cycles
{1}
{1,2}
{1,2,3}
87 clones
MAGE
9 cycles
96 clones
(No effect)
Millstone
+
elastic net regression
Top 8 candidate alleles
MAGE
9 cycles
96 clones
5 alleles
MAGE
7 cycles
359 clones genotyped to measure interactions among 6 alleles
6 final alleles
Generate New Ref. Genome
Model interactions between 6 final alleles
C321.ΔA.opt

Supplementary Figure 1

Detailed Experimental Workflow

Depiction of the specific steps used to identify the six alleles that optimized the fitness of C321.ΔA. *Millstone* (Goodman et al., submitted) was used to annotate mutations in C321.ΔA. 127 prioritized coding mutations were tested in C321.ΔA over 50 cycles of MAGE in 3 lineages. Eighty-seven clones were genotyped by whole genome sequencing and annotated using *Millstone*, and their doubling times were measured. Modeling by multiple linear regression identified 8 alleles for subsequent validation. After the second iteration, 5 alleles were chosen, with 3 alleles having a significant linear model coefficient and 2 more reversions having subtle effects. In a parallel experiment, a small pool of 7 non-coding mutations was tested, and modeling identified one allele that was found to have a strong effect. In another parallel experiment, 20 UAA-to-UAG reversions were tested on a C321 background with prfA still present, but no reversions were found to affect fitness. The top 6 alleles were combined in a final optimized strain, and clones with intermediate combinations of alleles were used to characterize their interaction effects (**Fig. 5**).

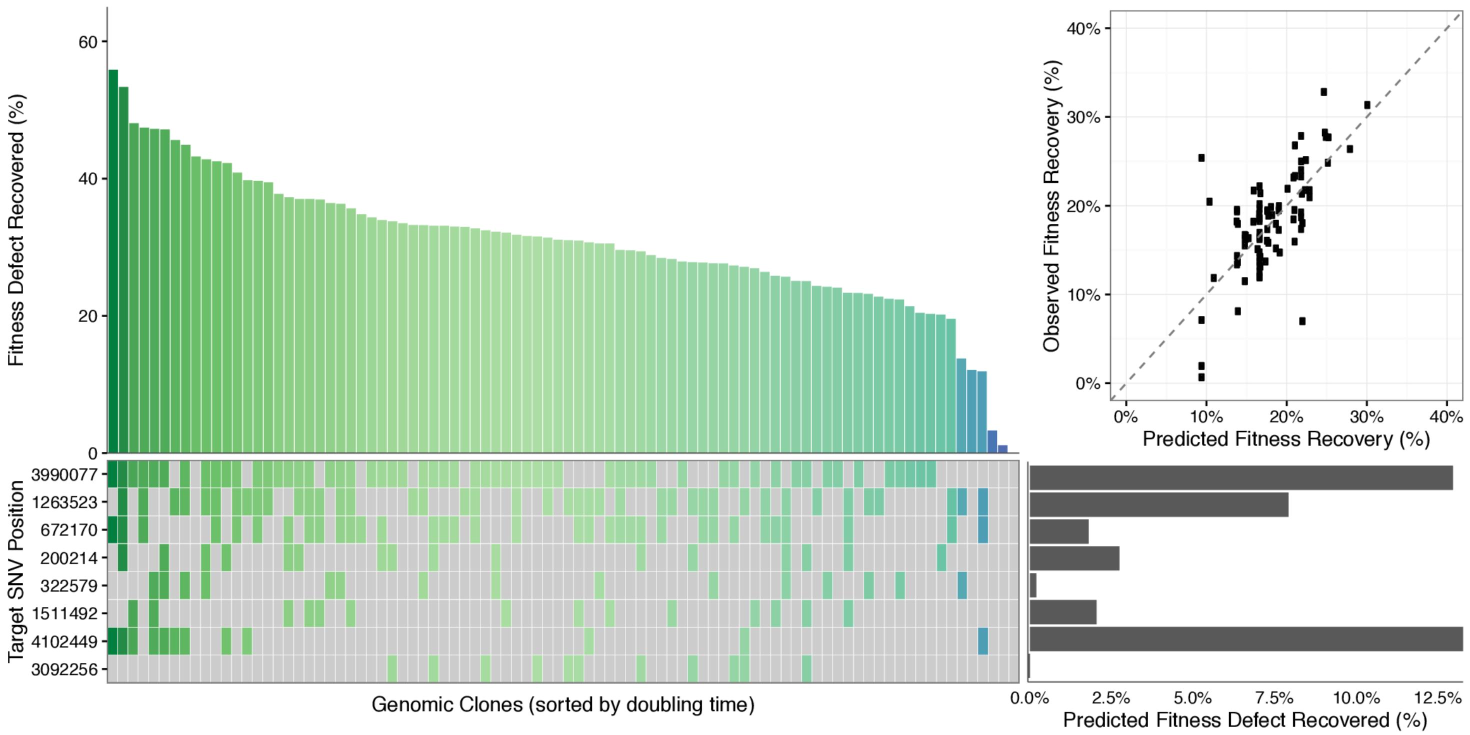

Supplementary Figure 2

Empirical testing to validate top eight alleles from 50-cycle MAGE experiment.

The top eight alleles (**Additional file 4**) were tested in the original C321.ΔA background using nine cycles of MAGE. We selected 96 clones from the final population and measured doubling times and performed MASC-PCR to assess genotypes. Clones are sorted by fitness on the x axis and alleles are listed on the y axis in order of enrichment. Linear modeling revealed a strong predicted effect for reversions *hemA*-T1263523C and *cpxA*-A4102449G and *de novo* mutation in *cyaA*-C3990077T, with weaker predicted effects for reversions *leuS*-C672170T and *bamA*-C200214T and *de novo* mutation T1511492C. For construction of the final strain, we chose to keep the three high-predicted-effect alleles (*hemA*-T1263523C, *cpxA*-A4102449G, *cyaA*-C3990077T) and the two weak-predicted-effect reversions (*leuS*-C672170T, *bamA*-C200214T), but we omitted the three weak-effect *de novo* mutations.

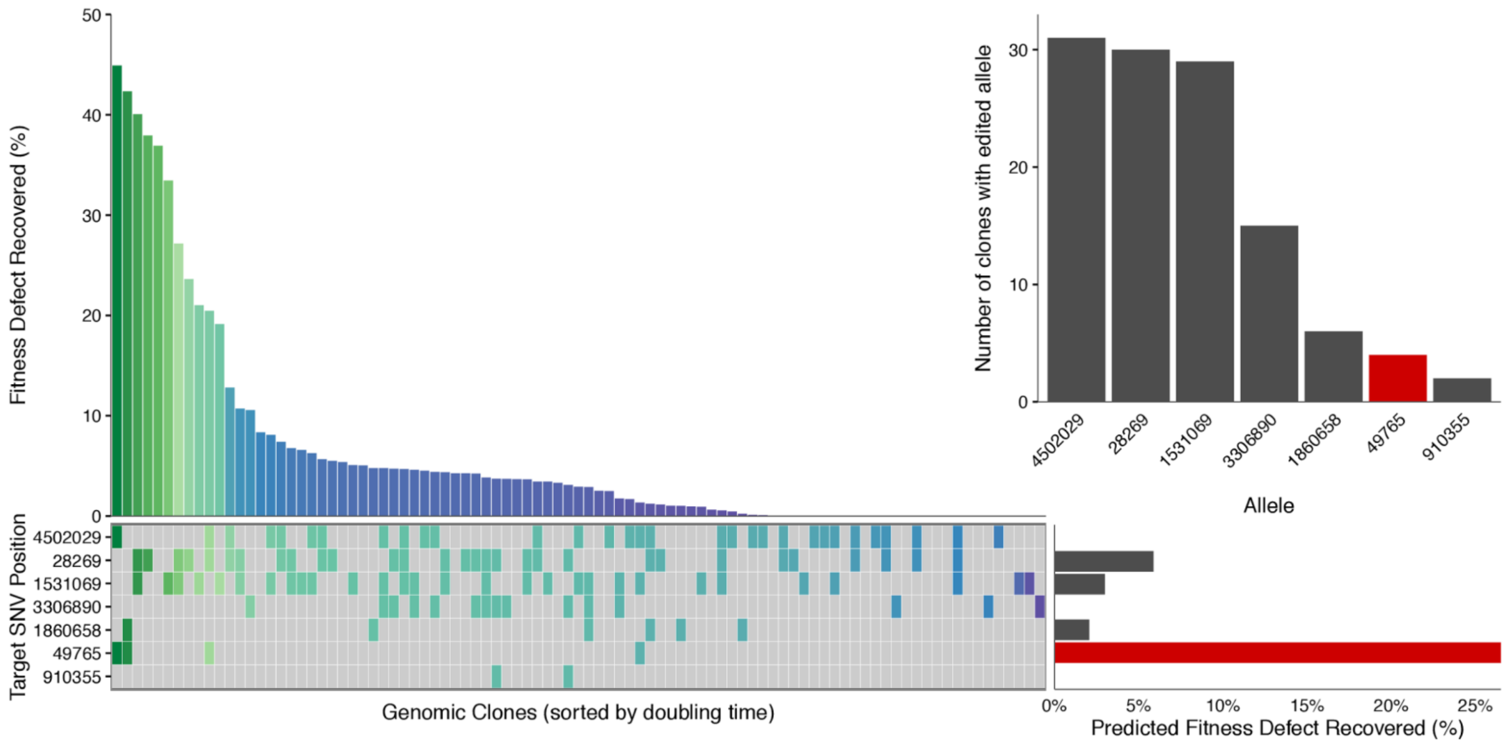

Supplementary Figure 3

Empirical testing identifies high-effect non-coding mutation.

Genotypes and fitness from testing a set of seven non-coding mutations (**Additional file 5**). Upper right: the top model-selected allele is not apparent from enrichment alone, and it is later experimentally validated to have a significant effect (**Fig. 4**).

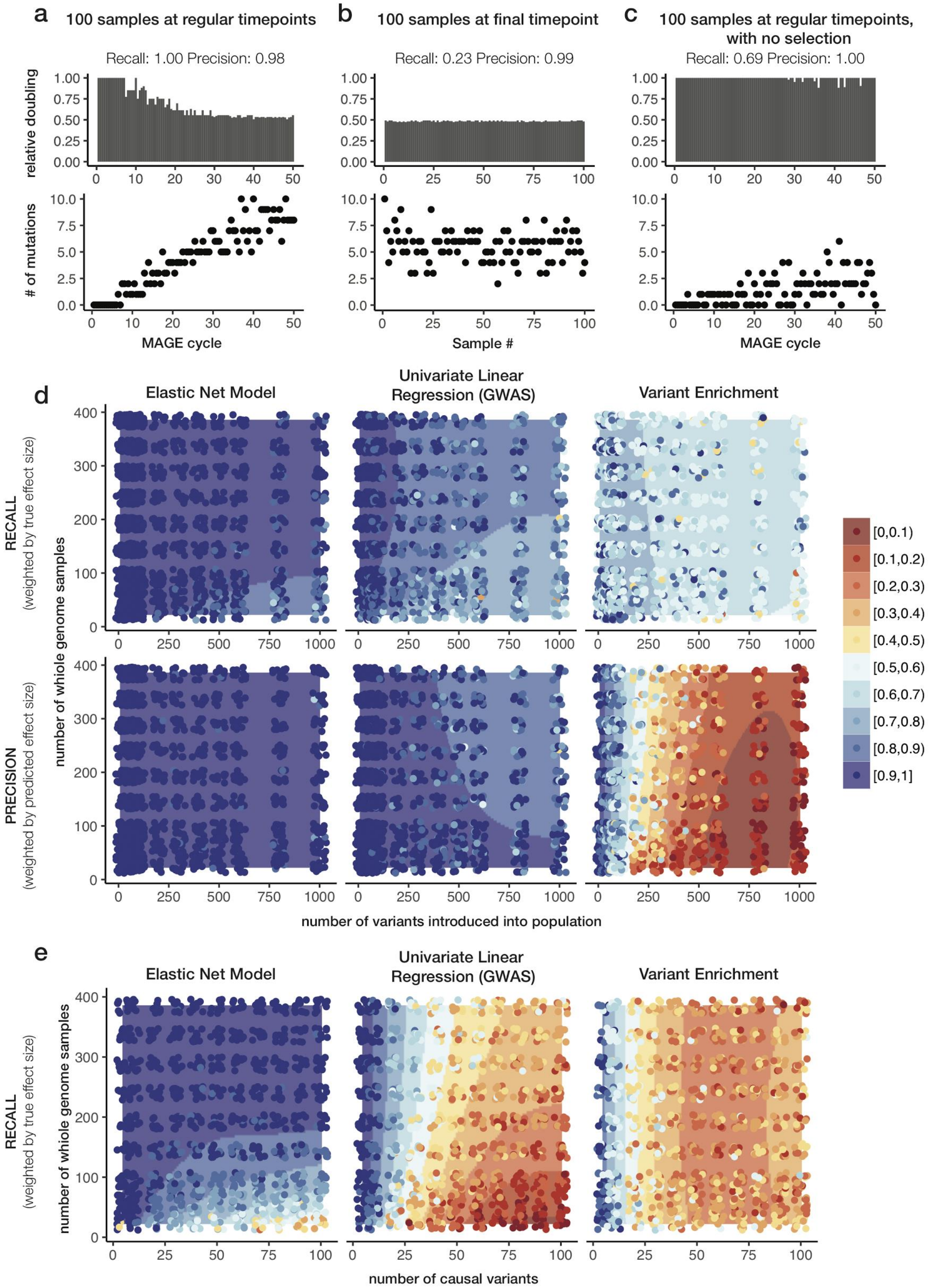
a
100 samples at regular timepoints
Recall: 1.00 Precision: 0.98
b
100 samples at final timepoint
Recall: 0.23 Precision: 0.99
c
100 samples at regular timepoints, with no selection
Recall: 0.69 Precision: 1.00
relative doubling
# of mutations
MAGE cycle
Sample #
MAGE cycle
d
Elastic Net Model
Univariate Linear Regression (GWAS)
Variant Enrichment
RECALL (weighted by true effect size)
PRECISION (weighted by predicted effect size)
number of whole genome samples
number of variants introduced into population
[0,0.1)
[0.1,0.2)
[0.2,0.3)
[0.3,0.4)
[0.4,0.5)
[0.5,0.6)
[0.6,0.7)
[0.7,0.8)
[0.8,0.9)
[0.9,1]
e
Elastic Net Model
Univariate Linear Regression (GWAS)
Variant Enrichment
RECALL (weighted by true effect size)
number of whole genome samples
number of causal variants

Supplementary Figure 4

Simulation illustrates predictive strength of elastic net model at different experimental design parameter combinations

Results of simulating varying experimental design parameters as described in **Supplementary Note 3**. (**a**) Doubling times and mutation distribution of clones sampled regularly over 50 cycles of simulated MAGE approximates what was observed in real data (**Fig. 2, Fig. 3**). (**b**) Sampling from final time point results in phenotypically homogenous population and reduced predictive modeling performance. (**c**) Running the simulation without selection results in insufficient propagation of effective alleles for predictive accuracy. (**d**) Precision and recall across different settings of the experimental design parameters of total variants introduced versus number of whole genome samples collected. A comparison among our elastic net linear modeling strategy and univariate linear regression (GWAS) and enrichment is shown. Points indicate results of simulation at corresponding parameters (10 replicates per parameter combo; separated by jitter). Fill color is interpolated using a LOESS regression. (**e**) Comparing number of genomes sampled vs number of underlying causal variants. Elastic net model predicted to achieve recall > 0.913 at at 200 whole genome samples for as many 100 causal effect SNPs.

Supplementary Notes

Supplementary Note 1

Further discussion of allele effect modeling and feature selection.

Adaptive laboratory evolution (ALE) typically uses enrichment of mutated genes observed across replicate lineages evolved in parallel to select meaningful features [33,34]. We selected multivariate linear modeling regularized by elastic net as an alternative to enrichment as initial tests suggested that it was a better method for predicting SNPs that recovered fitness in our experiments. For some alleles, high model coefficients corresponded to high levels of enrichment. For example, the reversion of mutation *hemA*-T1263523C had the highest enrichment after 50 cycles of MAGE (occurring in 78 out of 87 clones) and was also selected by modeling for validation, eventually being verified to confer fitness improvement (**Fig. 3**). On the other hand, when testing the pool of seven non-coding reversions, the single allele selected by the model *folA*-C49765T (also later experimentally validated) occurred in only 4 out of 96 clones. Meanwhile, three other alleles occurred in over 25 out of 96 clones, but they were not predicted to have a strong effect by linear modeling (**Supplementary Fig. 3**). There were many other cases where linear regression assigned low coefficients to alleles that were highly enriched. The discrepancy between enrichment and model-predicted effect may be due to differences in MAGE oligonucleotide recombination frequency [35], insufficient time for mutations to achieve enrichment, or stochastic enrichment of passenger mutations in a lineage during MAGE cycling. As noted in **Methods**, we observed that alleles with weaker effect were not consistently reported and were dependent on the choice of randomized train-test split, an issue that we expect to be remedied by sequencing additional clones and further tuning of the exact implementation of multivariate linear regression with respect to alleles with weak effect. Altogether, however, we found that regularized multivariate linear modeling provided an effective strategy of predicting fitness effect for individual alleles.

We compared our multivariate linear regression strategy to univariate linear regression (**Additional file 11**). Treating any Bonferroni-corrected p-value of < 0.05 as a reported effect, we found that the two modeling strategies agreed in six of the eight alleles chosen for validation following the 50-cycle MAGE experiment. Based on our simulations (**Supplementary Fig. 4**), we expect imperfect recall and precision for both elastic net and the univariate model. However, the elastic net model is predicted to generally perform better in both recall and precision, including at the parameters of our experimental design. Thus, we chose to pursue the targets identified by our elastic net regularized multivariate regression strategy. Further, the diminishing returns observed in our analysis of partial strains (**Supplementary Fig. 5**) suggests that the majority of effects had been recovered in the six SNPs found. We did not pursue independent validation of the additional mutations reported by the univariate analysis.

An important consideration with multivariate linear modeling is whether to include higher-order interaction terms. For our 50-cycle MAGE experiment, we made an assumption that independent allele effects would dominate relative to complex epistatic effects and included only first-order terms in the model. For validation in pools of <= 10 oligos, we typically selected 96 clones and experimented with second- and higher-order models, but also found that the first-order model was typically sufficiently informative of allele effect.

To investigate how higher-order model terms can inform interpretation of epistatic effects, we assembled a dataset of 359 intermediate genotyped clones obtained from validation experiments or from screening during construction of the final strain with all six top alleles (**Fig. 5**). Interestingly, linear modeling with second-order interaction terms indicated evidence of possible diminishing returns epistasis among certain alleles [36] and also a possible positive epistasis effect between *cpxA*-A4102449G and *cyaA*-C3990077T. Alleles that contribute to fitness through a positive epistasis effect could be lost during validation of small numbers of alleles, supporting our validation of high impact alleles in pools.

Even with targeted engineering by MAGE, *de novo* mutations can play a role in fitness improvement. The mismatch repair-deficient context in which this study was conducted elevates the background mutation rate >100-fold [37] and resulted in the accumulation of four *de novo* mutations for every reversion (**Fig. 2f**). We considered *de novo* mutations in modeling experiment data, but omitted any *de novo* mutation that was never observed in more than one clone, reducing the number of features corresponding to *de novo* mutations from 1329 to 135. Linear regression of data obtained over 50-cycles of MAGE identified four *de novo* mutations with a putative effect. Validation of these alleles determined that three of these were false positives or only beneficial in a specific context. The fourth *de novo cyaA*-C3990077T, however, showed a strong effect upon validation, demonstrating that linear regression can be an effective strategy for identifying causal *de novo* mutations and may be generally applicable in laboratory evolution studies.

Additional features beyond allele occurrence can be added to the linear model such as terms that capture prior expectation of an allele's effect. For application to ALE, mutations could be merged according to affected gene and its interacting partners. Higher order terms can be iteratively introduce as the candidate feature set is pruned.

Supplementary Note 2

Discussion of alleles chosen to construct final strain.

The final strain was constructed by introducing six mutations into the starting C321.ΔA background: five alleles reverted to their MG1655 starting point and one *de novo* mutation not previously present in MG1655 (**Additional file 7**).

Four of five reversions (*bamA*-C200214T, *leuS*-C672170A, *hemA*-T1263523C, *cpxA*-A4102449G) were coding mutations in essential genes prioritized in the highest category of 27 mutations in the 50-cycle MAGE experiment, supporting the strength of the initial prioritization method. The fifth reversion (p-*folA*-C49765T) was identified in screening noncoding off-target mutations predicted to disrupt gene regulation. Though we did not consider mutations outside of coding regions in our initial prioritization, the later consideration and validation of such a causal mutation demonstrates computational prediction of regulatory disruption as an important strategy in tuning organisms [8]. The sixth mutation was the *de novo* mutation C3990077T that arose in the background of MAGE-cycling, coding for *cyaA* (adenylate cyclase), a non-essential gene that nonetheless impacts fitness upon knockout (Keio knockout survival = 0.324). If designed, this mutation would have been prioritized in category 1 (**Additional file 2**).

We characterized intermediate genotypes created while constructing the final strain (**Fig. 5**) and determined that three of the mutations (reversions of p-*folA*-C49765T, *hemA*-T1263523C and *de novo cyaA*-C3990077T) had especially strong individual effects. Two reversions (*leuS*-C672170A, *bamA*-C200214T) had weaker individual effects that diminished in backgrounds with multiple mutations (**Fig. 5b**). The last reversion *cpxA*-A4102449G did not have a strong effect alone, and may even have been slightly detrimental, but appeared to provide a benefit in the presence of *cyaA*-C3990077T (**Fig. 5a**). To our knowledge, none of the gene identities or relationships reveal a first-order explanation for the findings of epistasis. However, both *cyaA* (C3990077T) and *cpxA* (A4102449G) are implicated in stress response pathways [38] [39].

We suspected that the *de novo cyaA*-C3990077T is a beneficial suppressor in the C321.ΔA background but not in the non-recoded background. Testing the mutation in EcNR1.mutS.KO revealed a minor detrimental effect on fitness, increasing doubling time by 2.94% (p=0.002; one-tailed t-test).

Supplementary Note 3

Simulating effects of experiment design parameters and modeling technique on power of predicting causal mutations.

Experimental design and modeling techniques affect the ability to detect causal alleles. In order to explore the effect of varying experimental parameters, we implemented an *in silico* simulation of MAGE cycling and predicted recovery of causal effects. Here, we describe the implementation of the simulation and insights learned about experimental parameters and comparisons of modeling methods. A Jupyter notebook containing the simulation code can be found at https://github.com/churchlab/optimizing-complex-phenotypes.

The simulation parallels our 50-cycle MAGE experiment and allows exploring the relationship among experimental design parameters including number of oligos tested and number of clones sampled for genotyping. The simulation also investigated different number and effect-size distributions of causal mutations. For a given combination of parameters, we sample a distribution of underlying mutation effects, which are distributed in effect size according to a power law distribution. The total fitness effect across all mutations is capped at a 50%, comparable to the C321.ΔA context. We then performed, *in silico*, iterations of MAGE separated by competitive expansion and bottlenecking of the population. We sample clonal genotypes from this simulated population and calculate phenotypes using the underlying mutation effects. We then perform predictive modeling with the simulated genotype-phenotype data and evaluate precision and recall relative to the true mutation effects. We also compare our regularized inear modeling strategy to univariate linear regression (as is used in GWAS) and enrichment of mutations in the final population. We made simplifying assumptions of no *de novo* mutations, no epistatic interactions among mutations, no measurement noise, and equal recombination efficiency for all mutations.

The simulations show that the predictive power of multivariate linear modeling requires a diverse set of genotype-phenotype pairs. Here, selection acts between MAGE cycles during expansion and bottlenecking of the population (re-growth to mid-log and sub-sampling for next round of MAGE). Sampling clones for genotyping at regular intervals over the course of MAGE cycling allows obtaining the needed genotype-phenotype diversity. We initially tuned the simulation parameters of recombination efficiency, distribution of fitness effects, and intervals between MAGE cycles by sampling clones until we observed fitness improvement and mutation accumulation distributions (**Supplementary Fig. 4a**) that were representative of our real data (**Fig. 2**). Sampling only from the final time point (**Supplementary Fig. 4b**), or simulating without selection (**Supplementary Fig. 4c**), resulted in a lack of phenotypic diversity and subsequently reduced predictive modeling power.

Using the simulations, we assessed the predictive capabilities of elastic net-regularized linear regression across different numbers of variants considered and number of whole genome samples, and compared our model to univariate linear regression (GWAS) and enrichment (**Supplementary Fig. 4d**). As evaluation metrics, we used variants of recall and precision that are weighted by underlying mutation effect and predicted mutation effect, respectively. We found that for a variety of parameter combinations, regularized linear modeling achieved higher recall and precision, and conclude that our approach yields better results than univariate regression or enrichment. By modeling the quantified effects of combinations of mutations, regularized linear modeling with elastic net can more effectively discriminate between causal mutations and hitchhikers. Our simulations also show that while recall decreases as the total

effect is distributed among greater numbers of individual mutations, it increases with the number of samples sequenced (**Supplementary Fig. 4e**). Sequencing up to 200 clones was simulated to capture at least 91% of causal effect for as many as 100 causal SNPs. Precision remains consistently high at different combinations of parameters.

We expect variations of our simulation strategy to be useful for design of other experiments and provide the simulation code on Github (https://github.com/churchlab/optimizing-complex-phenotypes).

Supplementary Note 4

Nucleotide sequences for 0-UAG-sfGFP, 1-UAG-sfGFP, 3-UAG-sfGFP used for characterizing nsAA incorporation.

>0-UAG-sfGFP
ATGCATCACCACCATCATCACAAAGGTGAAGAACTGTTTACCGGCGTTGTTCCGATCCTGGTTGAAC
TGGACGGTGACGTGAACGGTCATAAATTCTCCGTACGTGGTGAAGGTGAGGGTGACGCGACCAACG
GTAAGCTGACTCTGAAATTCATCTGCACCACCGGCAAACTGCCGGTTCCGTGGCCGACGCTGGTTA
CGACCCTGACCTACGGTGTTCAGTGCTTCGCGCGTTACCCGGACCATATGAAGCAGCACGACTTCTT
CAAATCTGCGATGCCGGAAGGTTACGTTCAGGAACGTACCATCTCTTTCAAAGACGACGGTACCTAC
AAAACCCGTGCGGAAGTTAAATTCGAAGGCGACACCCTGGTTAATCGTATCGAACTGAAAGGTATCG
ACTTCAAGGAAGACGGCAATATTCTGGGTCACAAACTGGAATACAACTTCAACTCTCACAATGTTTAC
ATCACCGCGGACAAACAGAAAAATGGTATCAAAGCAAATTTCAAAATCCGTCATAACGTTGAGGACG
GCTCTGTACAACTGGCGGACCACTACCAACAAAACACCCCGATTGGTGACGGTCCGGTCCTGCTGC
CGGACAACCATTACCTGTCTACCCAGTCTGTTCTGTCTAAAGACCCGAACGAAAAACGTGACCACAT
GGTTCTGCTGGAATTCGTTACCGCAGCGGGTATCACCCACGGTATGGACGAGCTGTATTAA

>1-UAG-sfGFP
ATGCATCACCACCATCATCACAAAGGTGAAGAACTGTTTACCGGCGTTGTTCCGATCCTGGTTGAAC
TGGACGGTGACGTGAACGGTCATAAATTCTCCGTACGTGGTGAAGGTGAGGGTGACGCGACCAACG
GTAAGCTGACTCTGAAATTCATCTGCACCACCGGCAAACTGCCGGTTCCGTGGCCGACGCTGGTTA
CGACCCTGACCTACGGTGTTCAGTGCTTCGCGCGTTACCCGGACCATATGAAGCAGCACGACTTCTT
CAAATCTGCGATGCCGGAAGGTTACGTTCAGGAACGTACCATCTCTTTCAAAGACGACGGTACCTAC
AAAACCCGTGCGGAAGTTAAATTCGAAGGCGACACCCTGGTTAATCGTATCGAACTGAAAGGTATCG
ACTTCAAGGAAGACGGCAATATTCTGGGTCACAAACTGGAATACAACTTCAACTCTCACAATGTTTAG
ATCACCGCGGACAAACAGAAAAATGGTATCAAAGCAAATTTCAAAATCCGTCATAACGTTGAGGACG
GCTCTGTACAACTGGCGGACCACTACCAACAAAACACCCCGATTGGTGACGGTCCGGTCCTGCTGC
CGGACAACCATTACCTGTCTACCCAGTCTGTTCTGTCTAAAGACCCGAACGAAAAACGTGACCACAT
GGTTCTGCTGGAATTCGTTACCGCAGCGGGTATCACCCACGGTATGGACGAGCTGTATTAA

>3-UAG-sfGFP
ATGCATCACCACCATCATCACAAAGGTGAAGAACTGTTTACCGGCGTTGTTCCGATCCTGGTTGAAC
TGGACGGTGACGTGAACGGTCATAAATTCTCCGTACGTGGTGAAGGTGAGGGTGACGCGACCTAGG
GTAAGCTGACTCTGAAATTCATCTGCACCACCGGCAAACTGCCGGTTCCGTGGCCGACGCTGGTTA
CGACCCTGACCTACGGTGTTCAGTGCTTCGCGCGTTACCCGGACCATATGAAGCAGCACGACTTCTT
CAAATCTGCGATGCCGGAAGGTTACGTTCAGGAACGTACCATCTCTTTCAAAGACGACGGTACCTAC
AAAACCCGTGCGGAAGTTAAATTCGAAGGCGACACCCTGGTTAATCGTATCGAACTGAAAGGTATCG
ACTTCAAGGAAGACGGCAATATTCTGGGTCACAAACTGGAATACAACTTCAACTCTCACAATGTTTAG
ATCACCGCGGACAAACAGAAAAATGGTATCAAAGCAAATTTCAAAATCCGTCATAACGTTGAGGACG
GCTCTGTACAACTGGCGGACCACTAGCAACAAAACACCCCGATTGGTGACGGTCCGGTCCTGCTGC
CGGACAACCATTACCTGTCTACCCAGTCTGTTCTGTCTAAAGACCCGAACGAAAAACGTGACCACAT
GGTTCTGCTGGAATTCGTTACCGCAGCGGGTATCACCCACGGTATGGACGAGCTGTATTAA

Supplementary Tables and Legends

Additional file 2 (Table S1): Prioritized coding reversion categories

Additional file 3 (Table S2): Table of 127 mutations targeted in the 50-cycle MAGE experiment
EFF_GENE: Affected gene, as reported by SnpEff.
EFF_TYPE: Effect of the mutation relative to wild-type MG1655, reported by SnpEff.
EFF_SEV: Severity scale, defined by SnpEff.
KEIO_LB_22HR: Growth rate relative to wild-type MG1655 under knockout of corresponding gene, as reported by [15].
KEIO_ESSENTIAL: Whether the gene is designated as essential for growth in [15].
ESSENTIALITY_MANUAL_CALL: Manual update of essentiality based on other resources, e.g. [40].
ESSENTIALITY_MANUAL_NOTES: Notes regarding ESSENTIALITY_MANUAL_CALL.
IS_RNA: Whether the mutation affects an RNA.
GO_TERMS: Associated gene ontology terms [41].
FROM_TOP_FITNESS: Whether target chosen from list of mutations with worst fitness effect.
FROM_TOP_EFF_SEV: Whether target chosen from list of mutations with top EFF_SEV.

Additional file 4 (Table S3): Top mutations from MAGE cycling

Additional file 5 (Table S4): List of non-coding mutations tested

Additional file 6 (Table S5): List of amber reversions tested

Additional file 7 (Table S6): List of top six mutations used to create C321.DA.opt

Additional file 8 (Table S7): List of additional mutations in C321.DA.opt

Additional file 9 (Table S8): Cost analysis

Additional file 10 (Table S9): Mutations from whole genome sequencing data for 90 clones from 50-cycle MAGE experiment
genomic_clone: Unique id corresponding to one of 90 clones sequenced following 50-cycle MAGE experiment.
*lineage***:** Which of the 3 lineages the this sample is from. NOTE: Lineage numbers here are 1, 3, 5 (corresponding to 1, 2, 3 in the text), and refer to the number of pooled libraries.
time_point: Sampling timepoint. See actual_mage_cycle.
*actual_mage_cycle***:** MAGE cycle corresponding to timepoint for this lineage.
*AF***:** Alternate frequency reported by Freebayes in calling variant. 1.0 = high-evidence alternate. 0 = strong-evidence wild-type (**Methods**).
GT_TYPE: Genotype called by Freebayes. 0 = wild-type, 1 = marginal, 2 = strong alternate (**Methods**).
*INFO_EFF_******: SnpEff annotations. See SnpEff documentation.

signal_relative_to_C321: 0 = same as C321. 1 = reverted to MG1655 allele or *de novo* mutation.
sum_signals: Sum of signal_relative_to_C321 across all clones for this variant.

Additional file 11 (Table S10): Comparison to univariate model (GWAS)
measured_effect: Experimentally-measured effect.
effect_pval: p-value of the experimentally-measured effect.
univar_pval: p-value of coefficient reported by univariate linear regression. Table is sorted by this value.
univar_est: Model coefficient reported by univariate linear regression.
elastic_net_coeff: Model coefficient reported by our elastic net-regularized multivariate regression method.
type: Whether the mutation is a designed reversion or *de novo* acquired during MAGE cycling.
